# An approach to avoid phrenic nerve injury during epicardial VT ablation

**DOI:** 10.21542/gcsp.2022.19

**Published:** 2022-12-30

**Authors:** Bart A. Mulder, Theo J. Klinkenberg, Yuri Blaauw

**Affiliations:** University of Groningen, University Medical Center Groningen, Department of Cardiology, Groningen, The Netherlands

## Abstract

Elimination of ventricular tachycardia with epicardial substrate may be limited due to close proximity of the phrenic nerve. In this case report we illustrate the use of a decapolar catheter in a Foley catheter for deviating the prenic nerve to safely perform epicardial ventricular tachycardia ablation.

## Introduction

Pathogenic mutations of the phospholamban (PLN) gene are common in the Netherlands and may give rise to highly arrhythmogenic cardiomyopathies^1^. Patients are at high risk for sudden death and ventricular tachyarrhythmias (VT) occur frequently^1^. Epicardial scar formation is predominantly found at the basal inferolateral left ventricle. Ablation of VTs originating from this area require an epicardial approach, but ablation can be hampered by close proximity of the phrenic nerve (PN)^2^. Several methods have been described for PN displacement, techniques including fluid overload or vascular or oesophageal balloon inflation^3^. However, it remains difficult to manoeuvre the balloon pericardially. We present our approach for PN displacement by using a Foley catheter.

### Case

**Figure 1.**
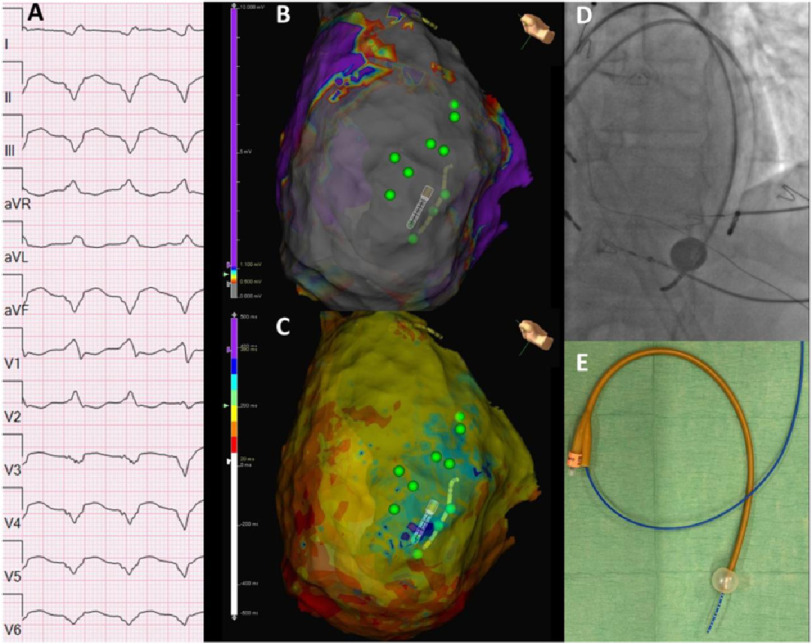
Foley catheter for phrenic nerve deviation during epicardial VT ablation. Panel A shows the clinical VT. Panel B shows the voltage map with a large area of scar (<0.500 mV) apical lateral. Green dots indicate phrenic nerve. Panel C shows very late activation (purple) and areas of slow conduction (crowding of isochrones) in the area of the phrenic nerve. Panel B and C both also show the epicardial position of the decapolar and ablation catheter. Panel D shows a LAO projection of the ablation catheter and the decapolar catheter within the inflated Foley catheter. Panel E shows the decapolar advanced through the Foley catheter.

A 63-year-old male patient with PLN cardiomyopathy (R14del) presented with electrical storm (multiple ICD therapies) despite using amiodarone (Figure 1A). Through a 2 cm subxiphoid incision direct pericardial access was obtained allowing introduction of materials. An extensive lateral scar with central slow conduction zones was observed. Pacemapping identified the isthmus of the dominant VT which was situated underneath the PN (Figure 1B–C).

We used a Foley catheter to deviate the PN from the ablation site (Figure 1D–E). A decapolar steerable catheter was introduced in the lumen of the Foley catheter. The small pericardial opening allowed easy access of both the Foley/decapolar catheter and the ablation catheter. The Foley/decapolar and ablation catheter were positioned at respectively PN and ablation location. Subsequently the balloon of the Foley catheter was inflated to deviate the PN and extensive ablation was safely performed in the area of the substrate (Figure 1B–D). Additional testing of phrenic nerve capture was performed before each lesion. Afterwards the clinical VT was non inducible. After two years of follow-up the patient did not experience any VT (or complication).

To our knowledge, this is the first report of the combined use of a decapolar catheter in a Foley catheter for deviating PN to safely perform epicardial VT ablation.
